# A Comprehensive Dynamic Life Cycle Assessment Model: Considering Temporally and Spatially Dependent Variations

**DOI:** 10.3390/ijerph192114000

**Published:** 2022-10-27

**Authors:** Shu Su, Jingyi Ju, Yujie Ding, Jingfeng Yuan, Peng Cui

**Affiliations:** 1 Department of Construction and Real Estate, School of Civil Engineering, Southeast University, Nanjing 211189, China; 2 Department of Engineering Management, School of Civil Engineering, Nanjing Forestry University, Nanjing 210037, China

**Keywords:** dynamic life cycle assessment (DLCA), environmental impact assessment, spatiotemporal variations, time-dependent, space-dependent, buildings

## Abstract

Life cycle assessment (LCA) is a widely-used international environmental evaluation and management method. However, the conventional LCA is in a static context without temporal and spatial variations considered, which fails to bring accurate evaluation values and hinders practical applications. Dynamic LCA research has developed vigorously in the past decade and become a hot topic. However, systematical analysis of spatiotemporal dynamic variations and comprehensive operable dynamic models are still lacking. This study follows LCA paradigm and incorporates time- and space-dependent variations to establish a spatiotemporal dynamic LCA model. The dynamic changes are classified into four types: dynamic foreground elementary flows, dynamic background system, dynamic characterization factors, and dynamic weighting factors. Their potential dynamics and possible quantification methods are analyzed. The dynamic LCA model is applied to a residential building, and significant differences can be observed between dynamic and static assessment results from both temporal and spatial perspectives. This study makes a theoretical contribution by establishing a comprehensive dynamic model with both temporal and spatial variations involved. It is expected to provide practical values for LCA practitioners and help with decision-making and environmental management.

## 1. Introduction

Life Cycle Assessment (LCA) is an international mature tool, which is widely used to assess environmental impacts and manage the environment. This approach follows the perspective of a whole life cycle, namely “from cradle to grave”, and has the advantages including systematicity, objectivity, openness, etc. The ISO issued a series of standards for LCA (i.e., ISO 14040–14044) [1], and many research institutions have developed mature LCA tools in the past several decades, such as GaBi [2], SimaPro [3], and OpenLCA [4]. LCA has been applied in manufacturing, agriculture, and construction industries to support design optimization, solutions comparison, decision-making, and so on [5].

The conventional LCA is a static assessment system, implicitly assuming that the resource consumption and pollutant emission levels, environmental background concentrations, and environmental effect mechanism remain unchanged during the whole assessment period. The environmental emissions/activities are usually aggregated into single values. In fact, many assessment parameters are temporal- and spatial-dependent. For example, the resource consumption and emissions occur at different time points, and the emission intensity of supply chain products may change due to technology development, and the characterization models for some impact categories vary in different regions [6,7]. The ISO 14042 standard clearly points out that the conventional LCA may weaken the environmental relevance of results [1]. In 1998, Herrchen firstly suggested to involve spatiotemporal dynamics in LCA research in a published paper [8]. Later, DLCA (Dynamic LCA) has gradually gained attention and become a research hotspot in recent years. DLCA is considered to be more effective and accurate [9], and can provide a new perspective for environmental impact [10]. Amounts of DLCA papers have been published in the past five years [11], and DLCA is regarded as a leading direction with potentials and challenges [12].

Some DLCA studies have been conducted, but most of them are fragmented ones with limited dynamic variations considered. Some studies only focused on dynamic variations in the temporal dimension. For example, Negishi et al. recognized several temporal factors of building systems, including insulation performance of walls, occupant behaviors change, energy mix evolution, and others [10]. Yang et al. considered temporal variations in electricity mix and steel manufacturing technologies in the carbon assessment of crop residue gasification projects [13]. Some scholars cared about spatial dynamics in LCA. Othoniel et al. used regional-specific characterization factors to provide a more representative evaluation of land use in Luxembourg [14]. Tabatabaie et al. focused on the differences in life cycle inventory databases in different regions [15]. There are limited studies systematically analyzing and quantifying both temporal and spatial variations.

Some scholars tried to develop dynamic models to provide assessment methodology. Levasseur’s research group focused on global warming impact and adopted integral calculations to aggregate temporal emissions and dynamic characterization factors [16,17]. Collinge’s group used dynamic matrices (Ct, Bt, At and ft) to reveal the time-dependent assessment process. The dynamic model was adopted to evaluate temporal ecological damages and human health impacts of buildings [18,19]. Su’s group analyzed the transformation pathway of calculation data, and then developed a temporally dynamic assessment model. Detailed steps and approaches for quantifying dynamic elements were analyzed, and some information technologies (e.g., BIM) were combined [20,21,22]. Negishi’s group proposed a five-step dynamic model with multi-scenario analysis of temporal parameters [10,23]. However, most available DLCA models are specialized only for temporal dynamics.

Although great and fast progress has been made in the DLCA research field, there are still some limitations. First, there are few studies systematically analyzing and quantifying spatiotemporal dynamic variations. Current DLCA studies just involved limited specific dynamic factors. Second, a comprehensive and operable DLCA model combining temporal and spatial variations is still lacking. The main assessment steps, data requirement, and quantification approaches are expected to be clear. To fill in these gaps, this study establishes a comprehensive spatiotemporal DLCA model. The involved dynamics are well analyzed and quantified. A building is taken as the application case to demonstrate the assessment steps in detail, and impact results are analyzed from both temporal and spatial dimensions.

## 2. Spatiotemporal DLCA Model

### 2.1. Assessment Framework

**Figure 1:**
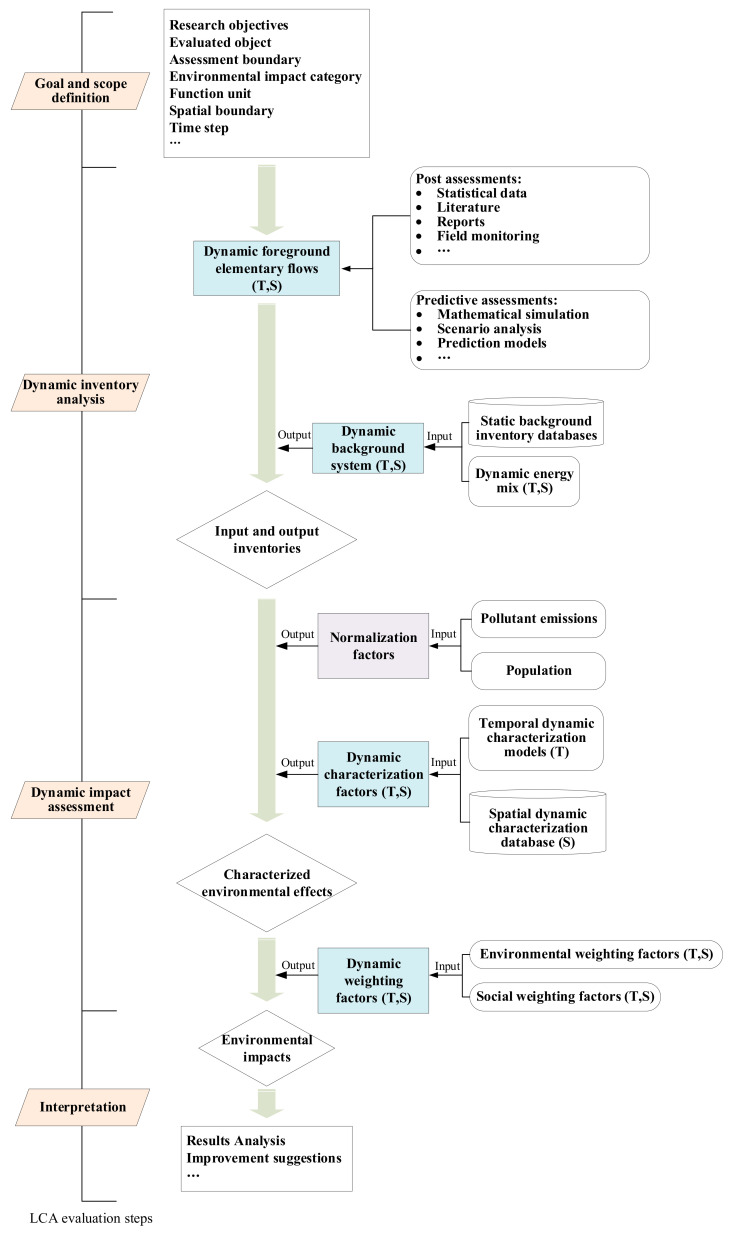
The spatiotemporal DLCA framework.

The spatiotemporal DLCA model is developed with temporal and spatial dynamic variations into consideration, as shown in Figure 1. The DLCA model still follows the standard paradigm of LCA (i.e., goal and scope definition → inventory analysis → impact assessment → interpretation). (1) In the goal and scope definition step, the research objectives, the evaluated object, assessment boundary, involved environmental impact categories, and functional unit are identified. In particular, the spatial boundary and time step are required to be clearly stated to support spatiotemporal evaluation. (2) In the dynamic inventory analysis step, the foreground elementary flows of the evaluated objects are collected. These flows are acquired and expressed in each time step and thus have temporal-dependent characteristics. The flows are spatial-dependent for local conditions and influences are considered. Then, these dynamic foreground elementary flows are transformed into raw material inputs and emission outputs by using spatiotemporal background inventory datasets. (3) In the dynamic impact assessment step, dynamic characterization factors are used to quantify the consequential environmental effects. A normalization step is required to convert the absolute impact values of various categories, which are generally expressed in different measurement scales, into normalized values. Currently, the majority of DLCA studies treat normalization as a static step, and no consensus on its temporal dynamics has been reached [24]. Thus, static normalization factors are used in this study. Finally, dynamic weighting factors are adopted to measure significances of different impact categories. (4) Interpretation is the last step, in which evaluation results are analyzed from multiple perspectives. Sensitivity analysis, uncertainty analysis, and scenario analysis may be conducted. Some improvement suggestions and schemes are provided for the evaluated objects according to the assessment results.

The calculation process and data transformation in DLCA can be expressed by five matrixes (i.e., dynamic foreground elementary flows, dynamic background datasets, dynamic characterization, normalization, and dynamic weighting), as shown in Equation (1). In this way, the possible spatial and/or temporal changes due to economic development, environmental change, and social progress during the evaluation period can be incorporated into assessment by evaluating their influences on the four dynamic matrixes. Thus, these four dynamic matrixes are the key to dynamic evaluation. The following sections will analyze their spatiotemporal dynamics and propose some potential approaches to quantify their dynamic variations:

(1) $$TEI\left(T,S\right)=\underset{n}{\sum }EI_{n}\left(T,S\right)=\underset{n}{\sum }EF_{i}\left(T,S\right)\times BI_{i-j}\left(T,S\right)\times CF_{j-n}\left(T,S\right)\times NF_{n}\times WF_{n}\left(T,S\right)$$

where TEI(T,S) is total environmental impact value of the evaluated object in year *T* and area *S* (dimensionless); $EI_{n}\left(T,S\right)$ is the value of *n*_th_ type impact category in year *T* and area *S* (dimensionless); $EF_{i}\left(T,S\right)$ is the amount of *i*_th_ type foreground elementary flow in year *T* and area *S* (kg); $BI_{i-j}\left(T,S\right)$ is background datasets of *i*_th_ type flow to *j*_th_ type raw materials/pollutant in year *T* and area *S* (kg/kg); $CF_{j-n}\left(T,S\right)$ is the characterization factor of *j*_th_ type raw material /pollutant to *n*_th_ type impact category in year *T* and area *S* (kg equivalent pollutant/kg); $NF_{n}$ is the normalization factor of *n*_th_ type impact category ([kg equivalent pollutant per capita]^−1^); and $WF_{n}\left(T,S\right)$ is the weighting factor of *n*_th_ type impact in year *T* and area *S* (dimensionless).

### 2.2. Dynamic Foreground Elementary Flows

#### 2.2.1. Dynamic Analysis

The foreground elementary flows include consumed resource and energy as well as discharged emissions, and they are case-specific and highly related to evaluated objects [25]. These flows usually occur in different processes and activities during the whole assessment period, and have typical temporal and spatial characteristics. The conventional static LCA studies usually regard the flows unchanged during the assessment period and aggregate temporal flows into single values, lacking temporal dimension. In addition, national data or industry average values are usually adopted in conventional LCA studies, insufficiently considering the spatial differences of environmental conditions, economic levels, technology, and others [26]. Thus, to improve the accuracy of evaluation results, spatiotemporal heterogeneity is suggested to be considered. 

The dynamic variations of foreground elementary flows vary product by product. For example, spatiotemporal variations of buildings’ operational flows include climate conditions, occupancy behaviors, thermal insulation properties of envelope, and others [23,27]. The spatiotemporal variations of energy crops include local temperature, humidity, planting slope, and soil property [28,29]. To select spatiotemporal variations, the following three characteristics are suggested to be considered. First, they are time and/or space-dependent; second, they have potentials to change during the evaluation period; and, third, their changes will have significant influences on the values of foreground elementary flows.

#### 2.2.2. Dynamic Quantification Methods

When transferring static foreground elementary flows into dynamic ones, researchers usually divided the whole assessment period into many smaller time steps and then collected local data at each time step to replace the conventional aggregated values. DLCA studies can be generally divided into post-assessment and predictive assessment ones according to the elementary flows occur after or before the evaluation point. Scholars adopted various methods to collect dynamic flows.

For post-assessments, dynamic foreground elementary flows already happened and can be acquired from existing accesses, such as statistics, literatures, and reports. For example, Bakas et al. obtained dynamic heavy metal concentrations in landfill leachate through literature [30]. Chen and Wang obtained resource consumption for pavement construction through reports by government agencies and academic institutions [31]. These data acquisition methods are very effort-saving, but data availability cannot be guaranteed. In addition, field monitoring is also an available data acquisition method. For example, Collinge et al. monitored building operational energy and indoor pollutant concentrations by several sensors [32]. Li et al. monitored emissions of suspended solids and phosphorus from wastewater treatment plants on a monthly basis [33].

In predictive assessment studies, simulations and predictions are needed to estimate the future elementary flows. Mathematical simulation, scenario analysis, and prediction models are common methods. (1) Mathematical simulation has the advantage of revealing real operational processes and helping to understand the nature of complex reality scenarios [34]. Komerska et al. used a software named DesignBuilder to simulate operational building energy consumption considering dynamic parameters of window-to-wall ratio, usable area, construction materials, and thermal properties [35]. (2) Scenario analysis estimates the expected values of an indicator considering possible occurrences, which is often used to describe the possible change scenarios of dynamic factors. For example, Ma developed two scenarios of energy mix in China during 2020 to 2030, and assessed the energy demands during this period [36]. (3) Prediction models are used to obtain possible future data according to historical data using a variety of vetted models and algorithms. For example, Cornago et al. predicted the hourly electricity mix in Italy with the deep neural network and then assessed the climate change impacts of an industrial plant [37]; Zhang et al. developed an energy consumption prediction model in an assessment of asphalt pavement [38]. The three methods above can well make up for missing data and predict future situations, but depend heavily on unfettered access to sufficient and accurate input parameters.

### 2.3. Dynamic Background Datasets

#### 2.3.1. Dynamic Analysis

Background inventory datasets are the collection of raw material and resource inputs as well as emission outputs per functional unit of products/services in the supply chain. Many institutions have established background inventory databases to provide data support, and the Ecoinvent from Switzerland, GaBi Databases from Germany, and NREL-USLCI from the America are typical and widely-used ones. The background inventory data have time-dependent characteristics. As a response, the Ecoinvent and GaBi databases are updated at intervals. Usually, the involved data in most databases are derived from industrial statistics and national reports without spatial characteristics. Recently, some scholars suggested to adopt local inventory datasets where the evaluated objects are located to improve the spatial resolution of results [39]. The latest version of Ecoinvent is improved to offer around 18,000 data items at three spatial scales (namely global, nation, and region) [40]. The inventory datasets of thermal power in the Chinese Life Cycle Database (CLCD) vary region by region [41].

Currently, the dynamic emission intensity of energy has received attention and been involved in many DLCA studies [42,43,44]. Energy is a necessity in the production and use of most products, and its inventory datasets have significant temporal and spatial dynamic characteristics. The technological advances over time in renewable energy are the main driving force of temporal dynamics. Taking the energy mix during 2015–2020 in China as an example, the proportion of coal falls from 64% to 57% while the shares of renewable energy increased from 17% to 25% [45]. The significant differences in resource endowment in different regions cause the spatial dynamics of inventory datasets of energy. For example, Shanxi province has abundant fossil energy while hydroelectric power is usually used for electricity generation in Sichuan province. The carbon emission factors of energy in the northeast and east of China are 1.0826 t CO_2_/MWh and 0.7921 t CO_2_/MWh, respectively [46].

#### 2.3.2. Dynamic Quantification Model

**Figure 2:**
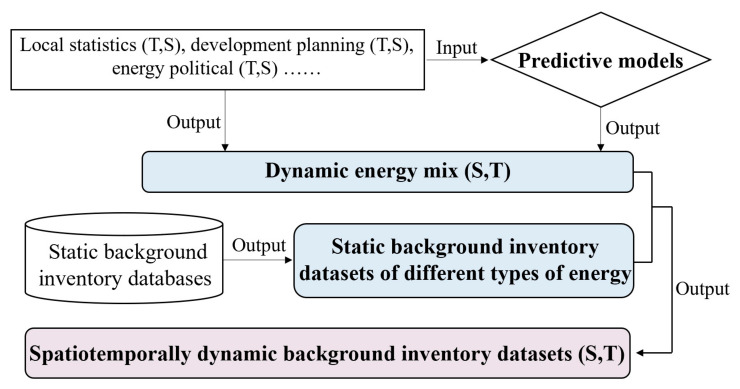
Dynamic quantification flowchart of the background inventory of energy.

Figure 2 shows the dynamic quantification flowchart of the background inventory of energy. Predictive models are adopted to simulate the dynamic energy mix levels during the whole assessment period based on historical data and development planning. The long-range Energy Alternatives Planning model (LEAP), back propagation model, and grey forecasting model are potential feasible models to conduct prediction. The input parameters are identified with local characteristics and temporal variations fully considered, and they can be derived from official statistics, development planning documents, and energy policies.

The predictive models output dynamic energy mix, reflecting the varying proportion of different energy types. Then, they are combined with static background inventory datasets of various types of energy to calculate spatiotemporal dynamic datasets of an integrated energy, as shown in Equation (2). The static background inventory data can be acquired from mature databases. The combination of static background inventory datasets and dynamic energy mix has been a common approach and already applied worldwide in the fields of buildings [19], energy policy [44] and agriculture [47], and so on:

(2) $$BI_{El-j}\left(T,S\right)=\sum_{k}P_{k}\left(T,S\right)\times BI_{k-j}$$

where $BI_{El-j}\left(T,S\right)$ is background inventory datasets of unit integrated energy to *j*_th_ type raw material/pollutant in year *T* and area *S* (kg/kg); $P_{k}\left(T,S\right)$ is the share of *k*_th_ type energy among the total energy in year *T* and area *S* (dimensionless); and $BI_{k-j}$ is the background inventory datasets of *k*_th_ type energy to *j*_th_ type raw material/pollutant (kg/kg). *k* represents energy types, including coal, hydro, nuclear, solar, and so on.

### 2.4. Dynamic Characterization

#### 2.4.1. Dynamic Analysis

Characterization factors quantify the potential environmental effects of various inputs and outputs, and they aggregate different effects of several pollutants into the environmental effect of the equivalent pollutant. Constant characterization factors are often adopted in conventional LCA studies, and the underlying assumption is that the environmental effects due to emissions distributed over the evaluation period are equal to the effects caused by the same amount of emissions happening at some time point. In reality, environmental effects are affected by the discharge points, emission rates, and environmental background concentrations [48]. For example, CO_2_ and CH_4_ are two types of greenhouse gases. The warming effects caused by CH4 sharply occur in the first few years while the effects caused by CO_2_ can last for hundreds of years [16]. Currently, several temporal characterization models of specific impact categories have been established. Levasseur et al. [16] and Ericsson et al. [49] used instantaneous radiative forcing and mean surface temperature indicators, respectively, to develop temporally dynamic characterization models for global warming impact. Lebailly et al. [50] and Shimako et al. [51] took dynamic fate factors and the USEtox^®^ model to calculate temporal dynamic characterization factors of ecotoxicity and human toxicity. In addition, dynamic characterization research on water resource, photochemical pollution, ozone depletion, and acidification have received some progress [11].

Traditional LCA assumes that the environmental effects of emissions are the same in different regions [52]. However, recent studies have shown that spatial heterogeneity exists. The local environment, background concentrations, resource availability, and concentration–effect relationship thresholds vary from region to region, affecting characterization levels [48]. Shah and Ries calculated the characterization factors of photochemical pollution in 48 states in the USA and showed significant differences [48]. Kounina et al. calculated characterization factors of toxic emissions at 0.5° × 0.5° resolution [53]. Núñez et al. calculated dynamic characterization factors of water use at 117 watersheds in Spain [54]. So far, some institutions have tried to establish spatial characterization databases to offer regional values. For example, IMPACT World + offers 44 characterization factors at four levels (namely global, continent, country, and native) [55]. Unfortunately, the above spatial dynamic characterization models lack a temporal perspective.

#### 2.4.2. Dynamic Quantification Method

**Figure 3:**
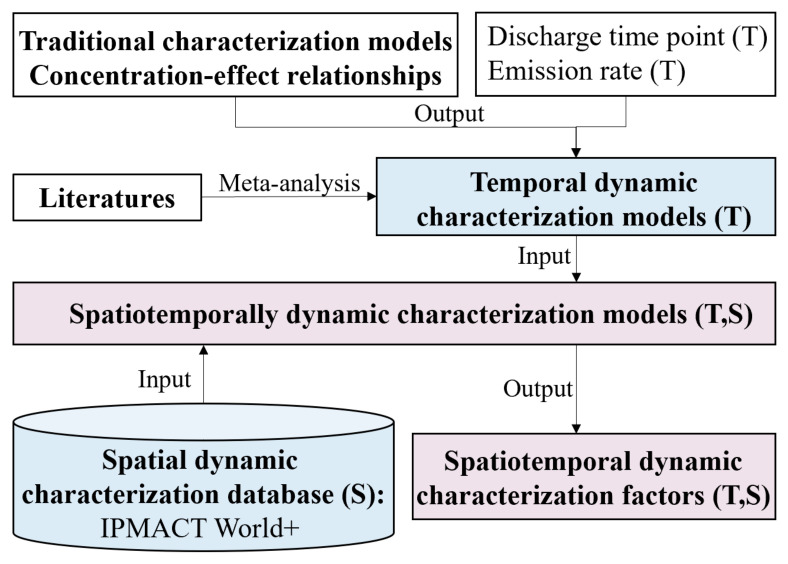
Dynamic quantification flowchart for establishing dynamic characterization factors.

Both spatial and temporal information are incorporated to establish dynamic characterization factors, and Figure 3 presents the research approach. The traditional static concentration–effect relationships and characterization models are used to reveal the mechanism between the exposure concentrations and environmental effects. Then, emission discharge times and discharge rates are combined to establish temporal characterization models. As another approach, meta-analysis can be adopted to directly gather available temporal characterization factors from literatures, which is a convenient and widely used method in current DLCA studies [56]. In the spatial dimension, IMPACT World+ is adopted to provide space-dependent factors. Combining temporal characterization models and spatial characterization factors, spatiotemporally dynamic characterization factors can be calculated.

### 2.5. Normalization and Dynamic Weighting

#### 2.5.1. Normalization

The characterized effects of different impact categories are generally presented in incompatible units, such as kg CO_2_-eq and kg SO_2_-eq. It is necessary to take preliminary steps to eliminate computational issues related to different measurement scales when more than one impact category is involved. The normalization step is used to transfer absolute values of different impacts into relative ones, enabling comparison, ranking, and aggregation [57]. Referring to conventional LCA studies [58], the characterized environmental effects in the base year for a given geographical area on a per capita basis are taken as the normalization reference indicator in this study, as calculated in Equation (3):

(3) $$NF_{n}=\frac{1}{\frac{\sum_{j}pol_{j}\times CF_{j-n}}{p}}=\frac{p}{\sum_{j}pol_{j}\times CF_{j-n}}$$

where $NF_{n}$ is normalization factor of *n*_th_ type impact category ([kg equivalent pollutant]^−1^); $CF_{j-n}$ is characterization factor of *j*_th_ type pollutant to *n*_th_ type impact category (kg equivalent pollutant/kg); $pol_{j}$ is the amount of *j*_th_ type pollutant (kg); and p is population.

#### 2.5.2. Dynamic Analysis of Weighting

The weighting system essentially quantifies the significances of different impact categories, reflecting the public’s acceptance of a comfortable environment and attitude towards environmental crisis to some extent [59]. Related values are usually constant in conventional LCA studies. In fact, public consciousness and political efforts may vary over time due to economic development, technological improvement, environmental priorities change, and others [60]. Therefore, temporal variations in weighting system are worthy of attention, and some scholars have already conducted some studies. Zhang established temporal cost functions of greenhouse gases and calculated dynamic weighting factors for 50 years [61]. Su et al. used the future pollutant reduction targets and resource usage planning indicators to develop a dynamic weighting system for ecological damage and resource depletion impacts [62].

On the other hand, geographic resource reserves and environmental conditions are various in different regions. More and more scholars suggested to incorporate spatial-specific information into a weighting system by using local parameters, such as population density [63] and resource costs [64]. Zhang et al. used local environment management policies and environmental carrying capacity to develop space-dependent weighting factors [65].

#### 2.5.3. Dynamic Weighting Model

**Figure 4:**
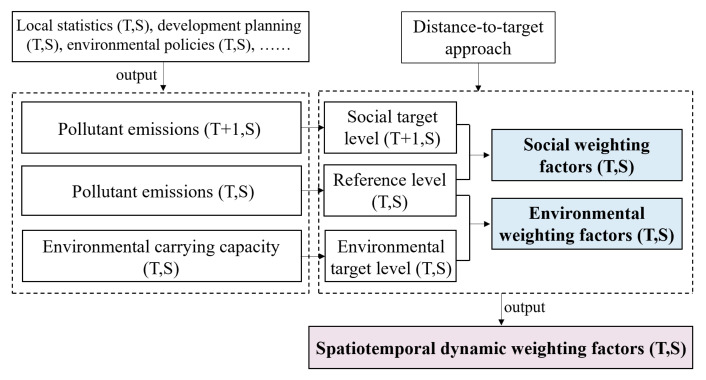
The dynamic model for spatiotemporal weighting factors.

Based on the authors’ previous studies [62,65], this study adopts a distance-to-target approach to develop spatiotemporal dynamic weighting factors. The distance-to-target approach measures the importance of an impact category by quantifying the distance from an existing situation (i.e., reference level) to a desired state (i.e., target level) [66]. As shown in Figure 4, the spatiotemporal dynamic weighting factors are calculated by combining social weighting factors and environmental weighting factors.

The social weighting factors reflect the temporal social attitudes to environmental damages, and its value in year *T* is calculated by the quotient of related environmental effect in year *T* and the environmental effect in the next year (as shown in Equation (4)). If the quotient is larger than 1, the reference level in current year is larger than the target level in the next year. This means that more efforts should be made to reduce emissions in the coming year, thus related environmental issues are more important:

(4) $$swf_{n}\left(T,S\right)=\frac{\sum_{j}pol_{j}\left(T,S\right)\times CF_{j-n}}{\sum_{j}pol_{j}\left(T+1,S\right)\times CF_{j-n}}$$

where $swf_{n}\left(T,S\right)$ is social weighting factor of *n*_th_ type impact category in year *T* and area *S* (dimensionless); $CF_{j-n}$ is characterization factor of *j*_th_ type pollutant to *n*_th_ type impact (kg equivalent pollutant/kg); $pol_{j}\left(T,S\right)$ is amount of *j*_th_ type pollutant in year *T* and area *S* (kg).

To consider spatial heterogeneity, the local environmental carrying capacity indicator is introduced to establish environmental weighting factors. The environmental carrying capacity reflects the ability of accommodating pollutants in a certain region. Once the discharged emission amounts exceed the local carrying capacity, this area will be in the overload state, which deserves more attention. Equation (5) presents the calculation of environmental weighting factors. The larger the value, the higher risk of environmental pollution overload, and the more important the corresponding impact category:

(5) $$ewf_{n}\left(T,S\right)=\frac{\sum_{j}pol_{j}\left(T,S\right)\times CF_{j-n}}{\sum_{j}ecc_{j}\left(T,S\right)\times CF_{j-n}}$$

where $ewf_{n}\left(T,S\right)$ is an environmental weighting factor of an *n*_th_ type impact category in year *T* and area *S* (dimensionless); $CF_{j-n}$ is the characterization factor of *j*_th_ type pollutant to *n*_th_ type impact category (kg equivalent pollutant/kg); $pol_{j}\left(T,S\right)$ is the amount of *j*_th_ type pollutant in year *T* and area *S* (kg); $ecc_{j}\left(T,S\right)$ is environmental carrying capacity of *j*_th_ type pollutant in year *T* and area *S* (kg).

As shown in Equation (6), with social and environmental weighting factors, spatiotemporal dynamic weighting factors can be calculated. “Plus 1” and “minus 1” are introduced into the equation in the case that both social and environmental weighting factors are smaller than 1. Due to space limitation, please refer to literature [65] for more detailed explanations about the equation:

(6) $$WF_{n}\left(T,S\right)=\{\begin{array}{c} (swf_{n}\left(T,S\right)+1)\times \left(ewf_{n}\left(T,S\right)+1\right)-1\;\;\;\;\;if\;swf_{n}\left(T,S\right)<1\;and\;\;ewf_{n}\left(T,S\right)<1\\ swf_{n}\left(T,S\right)\times ewf_{n}\left(T,S\right)\;\;\;\;\;\;\;\;\;\;\;\;\;\;\;\;\;\;\;\;\;\;\;\;\;\;\;\;\;\;\;\;\;\;\;\;\;\;\;\;\;\;\;\;\;\;\;\;\;\;\;\;\;\;\;\;Others\;\;\;\;\;\;\;\;\;\;\;\;\;\;\;\;\;\;\;\;\;\;\;\;\;\;\;\;\;\;\;\;\;\;\;\; \end{array}$$

where $WF_{n}\left(T,S\right)$ is the weighting factor of *n*_th_ type impact in year *T* and area *S* (dimensionless).

## 3. Application

### 3.1. The Evaluated Object

A typical residential building is taken as a case to apply the developed spatiotemporal DLCA model. The building is a high-rise one with 18 floors, and it is in the commonly used structure form (i.e., frame shear wall). The total floor area of the building is 7344 m^2^, holding 72 households. The average area per household (around 91.8 m^2^) is close to the national one (92.8 m^2^) [67]. The operation phase of the residential building is taken as the assessment boundary in this case study because the operation phase is the longest one during the whole life cycle of buildings, and related environmental impacts largely contribute to the total impacts. This building has been in use since 2016, and the following 4 years (from year 2016 to year 2019) are taken as the evaluation period with annually as the time step. The impacts of global warming, acidification, eutrophication, and airborne suspended particles are assessed. Per unit floor area of the building (1 m^2^) is taken as the functional unit in this assessment.

To show spatial variability, the building is assumed to be located in two different cities in China: Guangzhou city in Guangdong Province and Nanjing city in Jiangsu Province. The straight-line distance between these two cities is nearly 1132 km. Guangzhou is a coastal cosmopolitan. It is in the south of China with a total area of 7434.40 square kilometers and 18.8706 million people. Nanjing is in the east of China with a total area of 6587.02 square kilometers and 65.8702 million people. In terms of climatic conditions, Guangzhou is warm and rainy, and it has small temperature differences throughout the whole year. In contrast, the four seasons are more distinctive in Nanjing, where it is cold and dry in winter and hot and rainy in summer. In addition, there are some differences in residents’ behavior, social culture, and resource endowment in these two cities.

### 3.2. Dynamic Assessment

The foreground elementary flows of the residential building mainly include operational electricity, liquefied petroleum gas, natural gas, and water resources. Energy consumption mainly comes from cooling, heating, lighting, cooking, and other household equipment such as televisions, computers, washing machines, and so on. Living water is generally used for drinking, bathing, washing, etc. in daily life. The annual per capita household resource consumption levels in two cities during 2016–2020 are from the statistical yearbooks [68,69], which are authoritative materials published by the local Bureau of Statistics with high authenticity and accuracy. It is assumed that there are three persons in each household. Thus, the dynamic foreground elementary flows of the evaluated building in two cities can be calculated, as shown in Table S1.

Considering that electricity takes the leading share of all operational consumptions, the case study only considers the spatiotemporal dynamics in the inventory data of unit integrated electricity. The temporal energy mix information in two cities is from statistical yearbooks (as presented in Table S2) [70,71], which are reliable authoritative access. The static background inventory datasets of different energy types are from the CLCD database, which was updated in the year 2020. This database is mature and can represent the average technical level of Chinese market. With these parameters, the dynamic inventory datasets of electricity in two cities can be calculated using Equation (1). The dynamic inventory datasets are adopted to transform the foreground elementary flows into input and output data.

The adopted characterization factors in this case study are static ones for the following considerations. Firstly, current characterization research has focused on global warming and toxicity impacts. Temporal changes for some other impact categories, such as eutrophication and airborne suspended particles, are not well studied. This case involves several impact categories, and the assessment results may be biased if dynamic characterization factors of partial impact categories are used. Secondly, research on space-dependent characterization in Chinese cities is very limited. No available data can be directly adopted. Thus, static mature characterization factors are used in this study, and related dates are shown in Table S3 [72]. Many DLCA studies adopted static characterization factors due to dynamic data unavailability, which is a common practice [73,74].

Population and pollutant emissions in two cities in the base year (i.e., 2016) are obtained from statistical yearbooks [68,69,75], and they are adopted to calculate normalization factors. To calculate dynamic weighting factors, the annual pollutant emissions during 2016–2019 in Guangzhou and Nanjing are acquired from local statistics [68,69,75], as summarized in Table S4. The local environmental carrying capacities are calculated according to national levels [76,77] and local population proportions. This calculation is based on a principle that every person should bear equal environmental damage [65], and the calculated results are offered in Table S5. With the above data, the spatiotemporal dynamic weighting factors are developed (as offered in Table S6), and the final impact results of the case building can be assessed.

### 3.3. Analysis of Results

#### 3.3.1. Spatiotemporal Analysis of Annual Impacts

**Figure 5:**
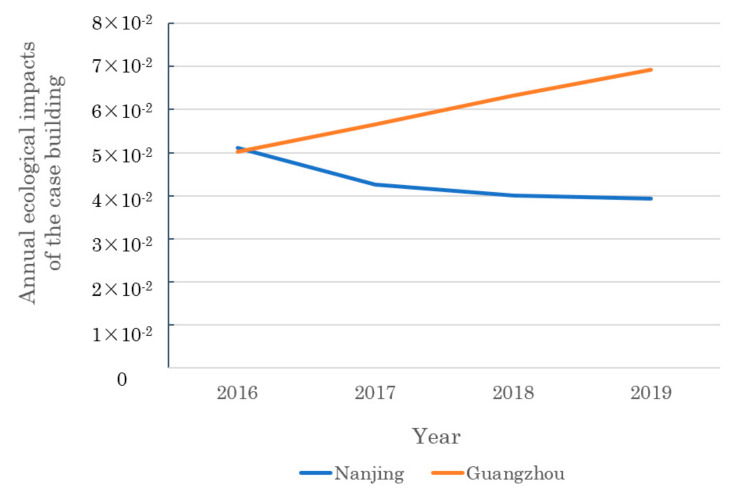
Annual ecological impacts of the case building (per m^2^) in Nanjing and Guangzhou.

Figure 5 shows the annual ecological impact results (per m^2^) of the evaluated residential building in two cities from 2016 to 2019. From the temporal perspective, the overall tendency of impacts in Guangzhou is obviously increasing, while that in Nanjing is moderately decreasing. This is because the dynamic energy mix was getting greener in Nanjing than Guangzhou. In addition, the dynamic weighting factors were decreasing in Nanjing due to greater emissions reduction, but are increasing in Guangzhou because more and more attention was paid to pollutants control. The peak values occur in 2016 in Nanjing (5.11 × 10^−2^) and happen in 2019 in Guangzhou (6.93 × 10^−2^). The largest annual impact difference reaches 1.19 × 10^−2^ in Nanjing and 3.11 × 10^−2^ in Guangzhou. Thus, temporal assessment provides a new and development perspective for the environmental impacts.

From the spatial perspective, the impacts in Nanjing have been lower than those in Guangzhou since 2017. The differences are very significant and the largest value can reach 3.00 × 10^−2^, which happened in 2019. This is because the foreground operational consumption in Nanjing is relatively lower, which is roughly 70–80% of that in Guangzhou. In addition, the dynamic weighting factors in Nanjing are decreasing, as shown in Table S6, since more efforts are devoted to emissions reduction. Comprehensively affected by the above factors, the final impact results in Guangzhou are larger. It can be seen that, when the same building is located in different regions, the consequential environmental impact levels are obviously different. Therefore, it is of great significance to consider spatial differences in LCA studies.

#### 3.3.2. Comparison of Different Impact Categories

**Figure 6:**
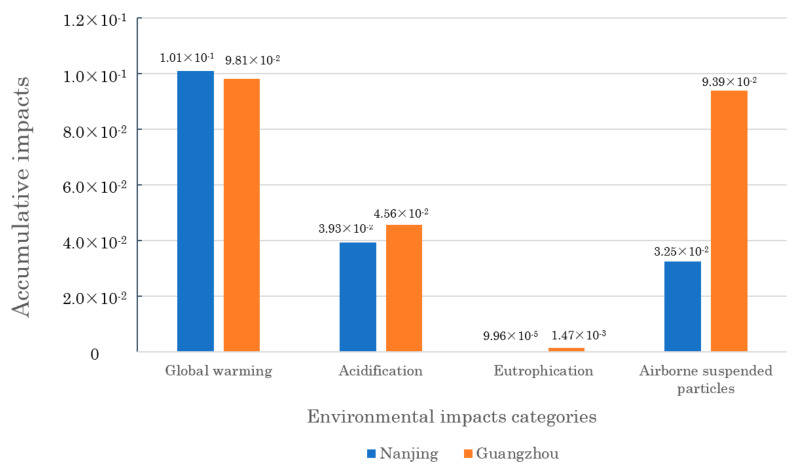
The accumulative impacts (per m^2^) of different impact categories during 2016–2019.

To compare the roles of different impact categories, Figure 6 presents the accumulative values (per m^2^) of different categories during 2016–2019. Global warming impacts in two cities make the leading contributions around 50%. This indicates that the greenhouse gases due to building operations are greatly concerning, and global warming is the most serious environmental problem. Eutrophication impacts play the smallest role, whose proportions among the total values are all less than 1% in two cities.

From the spatial perspective, the ecological issues of global warming and acidification in Nanjing are more serious than those in Guangzhou. However, the eutrophication and airborne suspended particles issues in Nanjing are relatively mitigatory. The two cities face different environmental problems and adopt different governance priorities.

## 4. Discussion

### 4.1. Contribution Analysis of Dynamic Element Type

**Figure 7:**
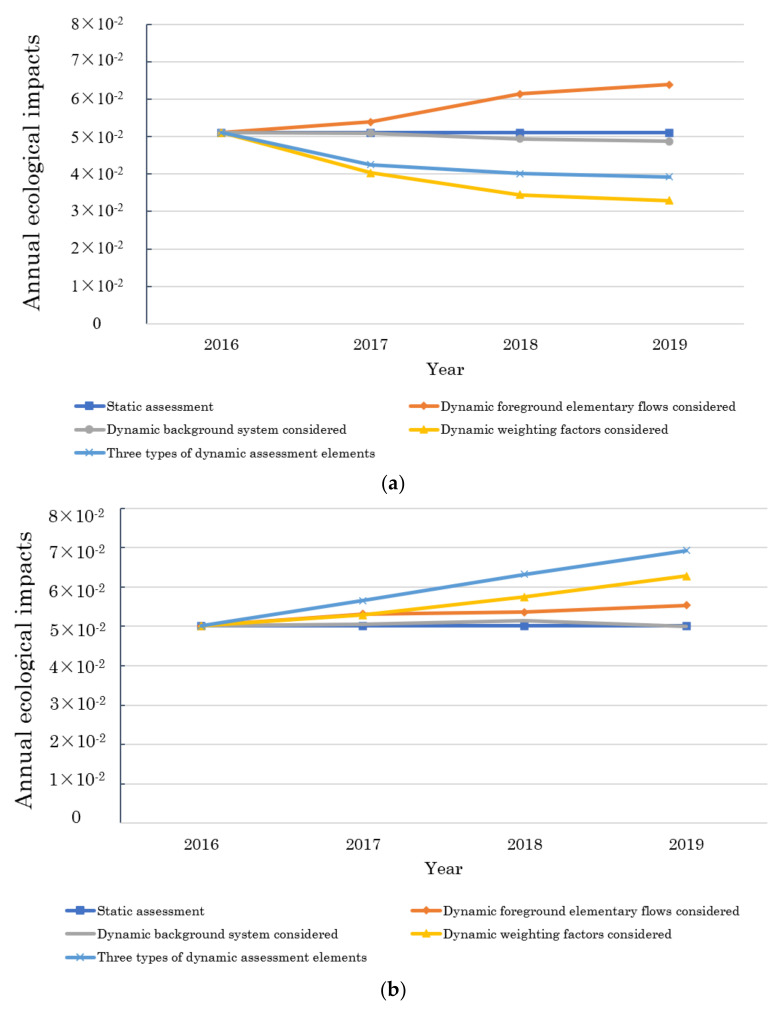
The annual ecological impacts (per m^2^) in (**a**) Nanjing and (**b**) Guangzhou with different types of dynamic assessment elements considered.

In the application study, three types of dynamic elements (i.e., dynamic foreground elementary flows, dynamic background system, and dynamic weighting factors) are considered. This section quantifies the impact results that with different types of dynamic assessment elements considered to analyze their contribution roles. The annual ecological impacts (per m^2^) in various scenarios are presented in Figure 7. The static assessment results involve none dynamic variations, and the annual values are 5.11 × 10^−2^ in Nanjing and 3.81 × 10^−2^ in Guangzhou. Three types of dynamic elements are incorporated into the calculation one by one to substitute the corresponding static values.

Evaluation results show that three types of dynamic elements affect impact results positively or negatively at different degrees. In two cities, the contributions of dynamic foreground elementary flows and dynamic weighting factors are relatively large, while the effects of dynamic background datasets are the smallest.

Dynamic foreground elementary flows have positive effects on impacts during four years in two cities. This is because the operational consumptions increase in the evaluation period, placing growing burdens on the environment.

Dynamic background datasets positively affect impacts in Guangzhou, and negatively affect impacts in Nanjing. This is because that proportion of thermal power in Guangzhou increased during the assessment period, while fossil energy was gradually replaced by clean energy in Nanjing.

The effects of dynamic weighting factors are influenced not only by local environmental carrying capacity, but also by the strengths of environmental protection policies. The dynamic weighting system plays a negative role in impacts in Nanjing and a positive role in impacts in Guangzhou.

To sum up, the differences between dynamic and static evaluation results are comprehensively influenced by a combination of various dynamic elements. Although the types of dynamic elements involved in two cities are the same, their influence magnitudes and directions are significantly different. Dynamic elements are distinguished due to spatial variability, and considering local specifics in LCA research has indispensable values.

### 4.2. Sensitivity Analysis of Parameters

Several parameters are involved in the dynamic assessment, and this section developed a single-dimensional sensitivity analysis of these parameters to better estimate their effects. Table 1 summarizes the change rates of ecological impacts (per m^2^) in 2019 when the values of analyzed parameters are plus or minus 10%. In two cities, the biggest influence on ecological impacts comes from the target levels of pollutants (−22.610–10.844%), orderly followed by the local proportion of thermal power generation (−9.690–10.811%), local population (−10.152–9.615%), operational electricity consumption per capital (−9.720–9.720%), environmental carrying capacity of CO_2_ (−5.862–7.165%), and environmental carrying capacity of dust (−2.015–2.463%). Therefore, the values of these parameters have a greater impact on the results. In contrast, the sensitivities of some parameters are very small, such as the environmental carrying capacity of SO_2_, NOx, and COD. Spatial differences between two cities can also be observed in Table 1. The change trends of ecological impacts for some parameters (i.e., population, environmental carrying capacity, and emission targets) are converse in two cities.

### 4.3. Meanings of Spatiotemporal Dynamic Assessment

This study established a comprehensive dynamic assessment model with temporal and spatial variations considered. The application study shows obvious differences between dynamic and static assessment results. From the temporal perspective, the ecological impact in Nanjing in 2019 is 23.21% less than that in 2016, and the difference percentage is 38.16% in the context of Guangzhou. The temporal differences during 4 years are significant, thus it can be inferred that the gap may be much larger when the assessment period is longer. Many evaluated objects such as buildings, bioenergy system, and agriculture have very long-life cycles, nearly decades. It is important and valuable to consider temporal variations in related assessment studies.

From the spatial perspective, the differences between annual ecological impacts in Nanjing and Guangzhou are dramatically obvious (1.86–76.56%). Although these two cities are located in the different parts of China, their spatial variabilities are relatively smaller when compared with those of cities in different nations or continents. Therefore, considering regional specifics and adopting local data are highly suggested in LCA studies. Conducting dynamic assessment can improve the spatiotemporal resolution and accuracy of assessment results, helping LCA practitioners to make better decisions. It takes a step forward when compared with the traditional static assessment.

## 5. Conclusions

This study proposes a spatiotemporal dynamic LCA model with four types of dynamic assessment elements considered (i.e., dynamic foreground elementary flows, dynamic background systems, dynamic characterization factors, and dynamic weighting factors). The dynamic variations in these four types of dynamic elements are analyzed and related dynamic quantification methods are put forward. A residential building that is assumed to be located in Nanjing and Guangzhou is taken as the case to demonstrate the applicability and steps of the developed dynamic model. Impact results are analyzed from temporal and spatial perspectives. Some useful conclusions can be drawn from the application and discussion:

(1) The proposed spatiotemporal DLCA model is operable and applicable.
(2) There are obvious differences between the temporal dynamic assessment results and static ones in the application case. Involving temporal variations in assessment studies for products with long-life cycles is meaningful, and can provide an evolutionary perspective.
(3) The spatial dynamic assessment results in two cities that are quite different. Considering regional specifics and adopting local data are highly suggested for LCA studies.
(4) The contribution of three dynamic element types to final results are quantified, and the influence directions and magnitudes depend on location and time.
(5) The sensitivities of involved parameters are various.

This study comprehensively analyzes the potential temporal and spatial dynamic variations embodied in LCA, and incorporates them to develop a spatiotemporal DLCA model. The main dynamic assessment steps, data requirement, and quantification approaches are fully discussed, deepening the theory of dynamic evaluation. The proposed dynamic model is testified to be operable in practice, and the assessment results have higher relevance and representativeness. The dynamic assessment is expected to bring more practical values, such as assisting better decisions and supporting reasonable environment management policies.

However, there is still room for improvement. Static characterization factors are used due to dynamic data limitation. Dynamic background inventory datasets are calculated by combining static background inventory datasets and dynamic energy mix. The involvement of static data may bring some uncertainties and decrease the accuracy of evaluation results to some extent. Improvements are suggested to be made in future studies.

## Figures and Tables

**Table 1:** Sensitivity analysis of parameters.

Parameters	Ecological Impacts (per m^2^) Change			
	10.000%		−10.000%	
	Nanjing	Guangzhou	Nanjing	Guangzhou
Electricity consumption per capital	9.649%	9.720%	−9.649%	−9.720%
Liquefied petroleum gas consumption per capital	0.017%	0.071%	−0.017%	−0.071%
Natural gas consumption per capital	0.142%	0.041%	−0.142%	−0.041%
Water consumption per capital	0.192%	0.168%	−0.192%	−0.168%
Proportion of local thermal power generation among total energy	10.881%	9.690%	−8.487%	−9.690%
Local population	−10.152%	−5.658%	9.615%	5.359%
Environmental carrying capacity of CO_2_	−5.862%	−3.268%	7.165%	3.994%
Environmental carrying capacity of dust	−2.015%	−1.523%	2.463%	1.861%
Environmental carrying capacity of SO_2_/ NO_x_/ COD	≈0.001%	≈−0.001%	≈0.001%	≈0.001%
Target levels of pollutants	−22.610%	−8.260%	10.844%	10.096%

## Data Availability

Not applicable.

## Supplementary Materials

The following supporting information can be downloaded at: https://www.mdpi.com/article/10.3390/ijerph192114000/s1, Table S1: The dynamic foreground elementary flows of the case building; Table S2: The temporal energy mix information in Jiangsu province and Guangdong province; Table S3: The adopted characterization factors of some environmental impact categories; Table S4: The annual pollutant emissions of some years in Guangzhou and Nanjing; Table S5: The environmental carrying capacities of emissions for Nanjing and Guangzhou; Table S6: The spatiotemporal dynamic weighting factors in Nanjing and Guangzhou during assessment period.
