# Supplementary material for: A Comprehensive Dynamic Life Cycle Assessment Model: Considering Temporally and Spatially Dependent Variations
Source: Int J Environ Res Public Health. 2022 Oct 27;19(21):14000. doi: 10.3390/ijerph192114000 (PMC9657249; doi:10.3390/ijerph192114000)
Supplement: Supplementary file 1 [file ijerph-19-14000-s001.zip › ijerph-1986771-supplementary.pdf]

**Supplementary Materials:**

Table S1. The dynamic foreground elementary flows of the case building

| | Nanjing | | | | Guangzhou | | | |
|---|---|---|---|---|---|---|---|---|
| | 2016 | 2017 | 2018 | 2019 | 2016 | 2017 | 2018 | 2019 |
| Electricity (kWh) | 1.81E+05 | 1.91E+05 | 2.17E+05 | 2.25E+05 | 2.19E+05 | 2.33E+05 | 2.36E+05 | 2.44E+05 |
| Liquefied petroleum gas (t) | 1.05E+03 | 9.72E+02 | 6.84E+02 | 6.46E+02 | 4.70E+03 | 4.18E+03 | 4.16E+03 | 3.69E+03 |
| Natural gas ($m^3$) | 8.82E+03 | 1.08E+04 | 1.18E+04 | 1.37E+04 | 3.99E+03 | 4.23E+03 | 4.44E+03 | 4.67E+03 |
| Water ($m^3$) | 9.46E+03 | 9.98E+03 | 1.18E+04 | 1.46E+04 | 1.35E+04 | 1.32E+04 | 1.35E+04 | 1.38E+04 |

Table S2. The temporal energy mix information in Jiangsu province and Guangdong province [62,63]

| Electricity generation capacity (kW) | Nanjing | | | | Guangzhou | | | |
|---|---|---|---|---|---|---|---|---|
| | 2016 | 2017 | 2018 | 2019 | 2016 | 2017 | 2018 | 2019 |
| Thermal power | 8.73E+07 | 9.44E+07 | 9.61E+07 | 9.85E+07 | 7.72E+07 | 7.39E+07 | 7.89E+07 | 8.52E+07 |
| Hydro power | 1.15E+06 | 2.60E+06 | 2.60E+06 | 2.60E+06 | 1.41E+07 | 1.08E+07 | 7.15E+06 | 9.42E+06 |
| Nuclear power | 2.12E+06 | 2.12E+06 | 4.37E+06 | 4.37E+06 | 9.38E+06 | 1.05E+07 | 1.33E+07 | 1.61E+07 |
| Solar power | 5.61E+06 | 5.21E+06 | 6.58E+06 | 8.53E+06 | 2.68E+06 | 2.91E+06 | 3.56E+06 | 3.89E+06 |

Table S3. The adopted characterization factors of some environmental impact categories [64]

| Impact category | Pollutants (kg) | Characterization factor |
|---|---|---|
| Global warming | $CO_2$ | 1 kg $CO_2$ -eq. /kg |
| | $CH_4$ | 25 kg $CO_2$ -eq. /kg |
| | CO | 3 kg $CO_2$ -eq. /kg |
| | $N_2O$ | 298 kg $CO_2$ -eq. /kg |
| Acidification | $SO_2$ | 1 kg $SO_2$ -eq. /kg |
| | $NH_3$ | 1.88 kg $SO_2$ -eq. /kg |
| | $NO_x$ | 0.7 kg $SO_2$ -eq. /kg |
| Eutrophication | $NO_x$ | 1.35 kg $NO_3^-$-eq. /kg |

| | | |
|---|---|---|
| | $NO_2$ | 1.35 kg $NO_3^-$-eq. /kg |
| | $N_2O$ | 1.41 kg $NO_3^-$-eq. /kg |
| | COD | 0.23 kg $NO_3^-$-eq. /kg |
| Airborne suspended particles | Dust | 1 kg /kg |

Table S4. The annual pollutant emissions of some years in Guangzhou and Nanjing [65][a] [60,61][b]

| Emission amount (million tons) | Nanjing | | | | Guangzhou | | | |
|---|---|---|---|---|---|---|---|---|
| | 2016 | 2017 | …… | 2020 | 2016 | 2017 | …… | 2020 |
| $CO_2$ eq. [a] | 8.59E+01 | 8.81E+01 | …… | 1.04E+02 | 8.02E+01 | 8.21E+01 | …… | 8.09E+01 |
| $SO_2$ [b] | 2.86E-02 | 1.54E-02 | …… | 9.70E-03 | 2.08E-02 | 1.54E-02 | …… | 4.00E-03 |
| $NO_x$ [b] | / | / | …… | / | 7.23E-02 | 1.92E-02 | …… | 7.10E-02 |
| Dust [b] | 4.86E-02 | 4.48E-02 | …… | 2.18E-02 | 1.29E-02 | 8.70E-03 | …… | 1.26E-02 |
| COD [b] | 9.00E-03 | 5.30E-03 | …… | 3.60E-03 | / | / | …… | / |

Table S5. The environmental carrying capacities of emissions for Nanjing and Guangzhou [66,67]

| Environmental carrying capacity (million tons) | Nanjing | | | | Guangzhou | | | |
|---|---|---|---|---|---|---|---|---|
| | 2016 | 2017 | 2018 | 2019 | 2016 | 2017 | 2018 | 2019 |
| $CO_2$ eq. | 9.42E+00 | 9.42E+00 | 9.44E+00 | 9.45E+00 | 1.73E+01 | 1.79E+01 | 1.84E+01 | 1.86E+01 |
| $SO_2$ | 7.20E-02 | 7.20E-02 | 7.22E-02 | 7.22E-02 | 1.32E-01 | 1.37E-01 | 1.40E-01 | 1.42E-01 |
| $NO_x$ | 7.69E-02 | 7.69E-02 | 7.71E-02 | 7.71E-02 | 1.41E-01 | 1.46E-01 | 1.50E-01 | 1.52E-01 |
| Dust | 3.08E+00 | 3.09E+00 | 3.09E+00 | 3.09E+00 | 5.67E+00 | 5.86E+00 | 6.01E+00 | 6.10E+00 |
| COD | 7.13E-02 | 7.14E-02 | 7.15E-02 | 7.15E-02 | 1.31E-01 | 1.36E-01 | 1.39E-01 | 1.41E-01 |

**Table S6. The spatiotemporal dynamic weighting factors in Nanjing and Guangzhou during assessment period**

| Impact category | Nanjing | | | | Guangzhou | | | |
|---|---|---|---|---|---|---|---|---|
| | 2016 | 2017 | 2018 | 2019 | 2016 | 2017 | 2018 | 2019 |
| Global warming | 8.88E+00 | 9.53E+00 | 8.84E+00 | 8.18E+00 | 4.85E+00 | 5.07E+00 | 4.61E+00 | 4.84E+00 |

| | | | | | | | | |
|---|---|---|---|---|---|---|---|---|
| Acidification | 7.38E-01 | 2.66E-01 | 2.03E-01 | 1.57E-01 | 4.09E-02 | 3.33E-01 | 4.82E-02 | 2.81E-02 |
| Eutrophication | 3.19E-03 | 1.36E-03 | 1.14E-03 | 6.71E-04 | 2.35E-01 | 1.82E-02 | 1.49E-02 | 2.53E-01 |
| Airborne suspended particles | 4.54E-01 | 3.50E-01 | 2.91E-01 | 5.16E-01 | 3.38E-01 | 1.59E-01 | 9.94E-01 | 1.17E+00 |
